# Development of a Self-management and Peer-Mentoring Intervention to Improve Transition Readiness Among Young Adult Survivors of Pediatric Cancer: Formative Qualitative Research Study

**DOI:** 10.2196/36323

**Published:** 2022-08-03

**Authors:** Adrienne S Viola, Kristine Levonyan-Radloff, Margaret Masterson, Sharon L Manne, Shawna V Hudson, Katie A Devine

**Affiliations:** 1 Department of Pediatrics Rutgers Cancer Institute of New Jersey New Brunswick, NJ United States; 2 Department of Medicine Rutgers Cancer Institute of New Jersey New Brunswick, NJ United States; 3 Department of Family Medicine and Community Health Rutgers Cancer Institute of New Jersey New Brunswick, NJ United States

**Keywords:** self-management, peer mentoring, cancer survivorship, long-term follow-up care

## Abstract

**Background:**

Childhood cancer survivors require lifelong risk-based follow-up care. It should be noted that less than one-third of adult survivors of childhood cancer report any survivor-focused care, and fewer than 1 in 5 obtain risk-based follow-up care. It is thought that this may be due to inadequate transition readiness, including low levels of knowledge, skills, motivation, and resources to make the transition to independent self-management of follow-up care. Interventions that focus specifically on improving the transition from parent-managed to self-managed care are needed. Theory and prior research suggest that targeting self-management skills and using peer mentoring may be innovative strategies to improve transition readiness.

**Objective:**

This study aims to identify the content of a self-management intervention to improve transition readiness among adolescent and young adult (AYA) survivors.

**Methods:**

Intervention development occurred in 3 stages: formative research with AYA survivors to identify barriers and facilitators to obtaining risk-based survivorship care, content development using feedback from multiple stakeholders (AYA survivors, parents, and providers), and content refinement (usability testing) of the initial proposed educational modules for the program. Content analysis, guided by the social-ecological model of AYA readiness for transition, was used to identify themes and develop and refine the content for the intervention.

**Results:**

A total of 19 AYA survivors participated in the formative research stage, and 10 AYA survivors, parents, and health care providers participated in the content development and refinement stages. The major barrier and facilitator themes identified included knowledge of cancer history and risks; relationships with health care providers; relationships with family members involved in care; emotions about health, follow-up care, and transfer of care; and lifestyle behaviors and life transitions. These themes were translated into 5 self-management modules: understanding treatment history and the survivorship care plan, managing health care logistics and insurance, communicating with health care providers and family members involved in care, dealing with emotions, and staying healthy in the context of life transitions. Feedback from the key stakeholders indicated that the content was relevant but should include participative elements (videos and tailored feedback) to make the intervention more engaging. The AYA survivors were receptive to the idea of working with a peer mentor and expressed a preference for using SMS text messaging, telephone calls, or videoconference to communicate with their mentor.

**Conclusions:**

Incorporating AYA survivors, parents, and providers in the design was essential to developing the content of a self-management and peer-mentoring intervention. AYA survivors confirmed the important targets for the intervention and facilitated design decisions in line with our target users’ preferences. The next step will be to conduct a single-arm trial to determine the feasibility and acceptability of the proposed intervention among AYA survivors of childhood cancer.

## Introduction

### Background

Childhood cancer survivors are a growing population, with >500,000 in the United States [1,2]. As many as 67% to 95% of these survivors are at risk for developing chronic health conditions [3-6] as a result of cancer treatment; therefore, survivors require lifelong risk-based follow-up care to identify and treat late health effects based on the cancer treatment they received [7]. It should be noted that less than one-third of adult survivors of childhood cancer report any survivor-focused care and fewer than 1 in 5 obtain risk-based follow-up care [8,9].

The transition from pediatric to adult follow-up care is a critical period when many survivors are lost to follow-up [10]. This transition involves moving from parent-guided management to self-management of long-term follow-up care as survivors assume primary responsibility for tasks such as managing health records, making appointments, filling and taking prescriptions, and understanding late effects and how to follow-up with recommended screenings or treatments. This transition occurs during a critical developmental period when adolescents and young adults (AYAs) may be unaware of, or fail to recognize, their health risks and the need for regular follow-up [11]. Barriers to successful transition include survivors’ lack of knowledge of their diagnosis and treatment, cancer-related anxiety and other emotional concerns, other life stressors common among AYAs that are perceived as a greater priority (eg, education and career), and failure or inability to assume personal responsibility for their own health [3,5,7].

Transition readiness refers to the capacity of the AYAs and their support network to prepare for, and complete, the process of moving to adult-oriented care [12]. The social-ecological model of AYA readiness for transition (SMART) [12] identifies modifiable factors related to transition readiness, including knowledge of health history, risks, and needs; self-management skills and self-efficacy for managing care; beliefs and expectations regarding the transition process or adult-oriented care (such as the belief that the adult provider will not understand the patient’s needs); goals related to health transition; relationships with parents and providers; and psychosocial functioning of patients, parents, and providers (such as anxiety about the transition process or future health) [12].

To date, interventions targeting AYA survivors of childhood cancer have focused on educational conferences [13,14], providing survivorship care plans through mail [15] or through a mobile app [16], or SMS text messaging and a healthy peer navigator intervention [17]. These interventions have used technology to overcome common barriers faced by AYA survivors, such as geographic mobility, lack of time, competing priorities, and the relatively small numbers of survivors at single institutions [18]. However, there is a gap in interventions focusing specifically on the transition from parent-managed to self-managed care. Peer mentoring has been recommended as an innovative approach to facilitate health care transitions for AYA survivors [19]. Qualitative and quantitative studies suggest that AYA survivors want to discuss their medical care needs with other AYA survivors [20-22]. The Adolescent and Young Adult Oncology Progress Review Group has recommended the development of standardized peer-to-peer programs as a strategy for supporting the psychosocial needs of AYA patients with cancer and AYA cancer survivors [14].

### Objectives

The goal of this work was to develop an intervention to improve transition readiness among AYA survivors of childhood cancer that would address their unique psychosocial and support needs. The development process involved identifying user needs (formative research), evaluating and applying relevant theory, and iteratively producing and refining the intervention product (ie, usability testing). We used principles of user-centered design, which propose an iterative process that identifies the needs and requirements of end users (ie, AYA survivors) and incorporates their feedback at multiple points in the intervention development [23]. The goal was to create an intervention that better meets the needs and expectations of the ultimate users of that intervention. In this paper we describe the user-centered design process used to develop a web-based self-management and peer-mentoring program to improve transition readiness of AYA cancer survivors.

## Methods

### Program Development

**Figure 1:**
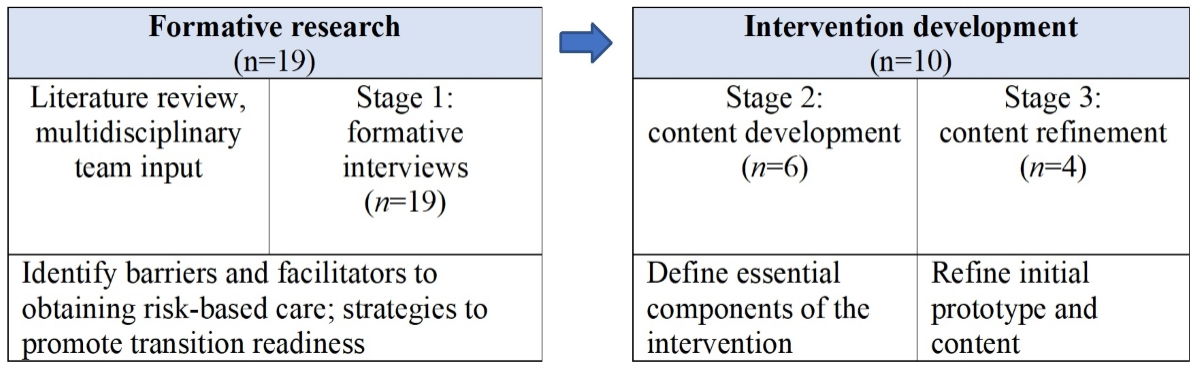
Managing Your Health intervention development stages.

Similar to other intervention development studies [24-26], we used an iterative user-centered process in three stages: (1) *formative research* with AYA survivors to identify barriers and facilitators to obtaining risk-based survivorship care during the transition to adult health care; (2) *content development* using feedback from multiple stakeholders (AYA survivors, parents, and providers); and (3) *content refinement* of the initial proposed content and educational modules for the program with input from AYA survivors (Figure 1). Semistructured telephone interviews were conducted with survivors in stage 1; semistructured in-person or telephone interviews were conducted with survivors, parents, and providers in stage 2; and in-person usability testing was conducted in stage 3.

### Participant Eligibility

Eligibility criteria were consistent across the stages. Survivors were eligible if they (1) were aged 18 to 25 years at the time of informed consent, (2) had been diagnosed with any cancer between the ages of 0 and 21 years, (3) were at least 1.5 years after completion of cancer treatment (consistent with preparing to transfer to long-term follow-up), and (4) had no physician- or self-reported cognitive delay or impairment that would prevent self-management of health care. Parents were eligible if (1) they were a primary caregiver of a pediatric cancer survivor currently aged 18 to 25 years, (2) the survivor was at least 1.5 years after completion of cancer treatment, and (3) the survivor did not have physician- or caregiver-reported cognitive delay or impairment that would prevent self-management of health care. Health care providers (eg, physicians and psychologists) were eligible if they regularly provided clinical care to AYA pediatric cancer survivors.

### Stage 1: Formative Research

The goal was to identify barriers and facilitators to obtaining risk-based survivorship care to understand the needs of AYA survivors transitioning from pediatric-oriented to adult-oriented care. AYA pediatric cancer survivors were recruited using advertisements posted on Twitter, web-based forums for cancer survivors, listservs of regional nonprofit organizations devoted to childhood cancer, and at the local clinic. This strategy was chosen to try to gain perspectives from survivors in various geographic locations who would represent a range of experiences with long-term follow-up care.

Individual semistructured interviews were conducted through telephone by the last author (KAD) and were audio recorded using digital recorders. Telephone interviews were chosen to allow participation from any geographic location and reduce respondent burden by not requiring an in-person visit. The semistructured interview guide was developed based on a model of factors associated with receipt of risk-based care [27] and the SMART model [12]. Questions elicited AYA survivors’ perspectives regarding survivor-related, provider-related, and health care system–related facilitators and barriers to receipt of care, including during the transfer from acute to long-term follow-up care and the transition from pediatric- to adult-oriented health care. Sample questions included the following: “What kind of follow-up care do you think is appropriate for cancer survivors? What care do you think you need? Tell me about the transition from the end of your cancer treatment to long-term follow-up care. What barriers could or do prevent you from attending care visits? What could clinics or providers do to encourage childhood cancer survivors to attend? If you could change something about follow-up care, what would you change?” Participants also completed a brief demographic survey.

Each interview was transcribed using semiverbatim transcription. Interview transcripts were analyzed using content analysis, following the framework approach described by Pope et al [28]. An index of themes was created and applied systematically to the data by 2 coders. Themes were identified from the a priori models, and any new themes that emerged from participants’ data were added during the coding process. Data analysis continued until theme saturation occurred (ie, no new themes emerged from additional participants).

### Stage 2: Content Development

The goal in this stage was to define the essential content components of the intervention. AYA survivors and caregivers were recruited from the local long-term survivorship clinic list. Health care providers (eg, physicians and psychologists) were recruited through email through the AYA and Survivorship Working Groups of the Children’s Oncology Group.

Semistructured individual interviews with AYA survivors, caregivers, and providers were conducted in person or through telephone and recorded for transcription and analysis. These interviews were guided by the SMART model and the results from the stage 1 formative research. The interview questions started by asking broadly about experiences with long-term follow-up care; for example, “What do you think is involved in the long-term follow-up care for your childhood cancer? What was challenging about beginning to take charge of your own health? What kinds of things were helpful to you in making the transition to taking charge of your health care?” Next, the themes from stage 1 were presented as the potential topics for a new intervention for survivors, and participants were asked to provide feedback on the proposed content. Questions included the following: “What seems useful to you? What does not seem useful? What are we missing?” The idea of matching survivors with a peer survivor who could serve as a mentor was proposed, and participants were asked about their receptivity to working with a mentor and preferred characteristics (eg, personality, cancer diagnosis, and gender). Participants were also asked to provide feedback about the mode of delivery (eg, web based, videoconference, or SMS text messaging). Qualitative data were analyzed on a continuing basis following the same procedures as those followed in stage 1 and treating respondents as a group of key stakeholders rather than examining them separately by type (ie, survivor vs parent vs provider).

### Stage 3: Content Refinement

AYA survivors were recruited from the local long-term survivorship clinic and completed the usability testing in person after their routine clinic visit. Participants were provided with the proposed structure of the program (ie, combination of web-based educational modules plus interaction with a peer mentor) and asked to review the prototype of web-based modules, which were accessible through Canvas, the Rutgers University web-based course management system, accessed through a computer or tablet provided by research staff. Survivors were asked to read through the program modules. Research staff asked questions about their overall impression of the program, the usability of the website, the content of each module, and suggestions for improvement. The staff asked survivors about their communication preferences (type of communication and frequency) and qualities they might prefer in a mentor. This feedback was then used to modify and finalize the intervention content and format.

### Ethics Approval

This study was approved by the institutional review boards at the University of Rochester Medical Center (RSRB00040459) and Rutgers Cancer Institute of New Jersey (Pro2013003819; Pro20150001955). All participants provided informed consent.

## Results

### Participants

In stage 1, a total of 19 AYA survivors from 12 different states in the United States participated. They were aged on average 22.8 (SD 1.6; range 20.0-25.0) years and were between 2.0 and 21.5 (mean 7.2, SD 6.2) years after completion of cancer treatment. In stage 2, a total of 6 key stakeholders participated: 2 (33%) AYA survivors, 1 (17%) parent of an AYA survivor, and 3 (50%) AYA oncology providers (of these providers, 1/3, 33% was an oncologist, and 2/3, 67% were psychologists). In stage 3, four new AYA survivors provided feedback on the initial prototype (Table 1).

**Table 1:** Adolescent and young adult survivor characteristics included in each stage of development.

		Stage 1: formative research (n=19)	Stage 2: content development (n=2)^a^	Stage 3: content refinement (n=4)
Age (years), mean (SD)		22.8 (1.6)	22 (1.4)	21.5 (1.5)
Sex, female, n (%)		17 (89)	2 (100)	1 (25)
**Race, n (%)**				
	White	18 (95)	1 (50)	1 (25)
	Asian	1 (5)	0 (0)	2 (50)
	Black	0 (0)	1 (50)	1 (25)
**Ethnicity, n (%)**				
	Hispanic or Latino	1 (5)	0 (0)	0 (0)
	Non-Hispanic	18 (95)	2 (100)	4 (100)
**Diagnosis, n (%)**				
	Blood cancer	15 (78.9)	1 (50)	4 (100)
	Solid tumor	3 (15.9)	1 (50)	0 (0)
	Brain tumor	1 (5.2)	0 (0)	0 (0)
**Currently receive follow-up care^b^, n (%)**				
	Yes	15 (78.9)	1 (50)	4 (100)
	No	4 (21.1)	1 (50)	0 (0)

^a^In stage 2, interviews were also conducted with 4 stakeholders (ie, n=1, 25%, parent of an adolescent and young adult survivor and n=3, 75%, adolescent and young adult oncology providers).

^b^This reflects whether participants reported receiving survivorship follow-up care but not the extent to which they have successfully transferred to adult-oriented care or managed their own care.

### Stage 1: Formative Research

In total, five themes were identified from survivors’ responses: (1) knowledge of cancer history and risks; (2) relationships with health care providers; (3) relationships with family involved in care; (4) emotions about health, follow-up care, and transfer of care; and (5) lifestyle behaviors and life transitions. Table 2 defines each theme, lists illustrative quotes, and provides examples of how the theme informed content development.

**Table 2:** Themes from the stage 1 formative research (N=19).

Theme	Definition	Illustrative quote	Translating into content
Knowledge of cancer history and risks	Lack of knowledge is a barrier to obtaining care. Having a written survivorship care plan and education about required ongoing care facilitates care	“I think it’s important to be knowledgeable about what you’ve been through and what could potentially happen. So I feel really lucky that I have that book [of my treatment summary and potential late effects] and I was educated.” [Female Hodgkin lymphoma survivor aged 23 years; 007] “I just want to control and see that everything is correct to get peace for my mind. But I usually go if I am starting to notice any signs what I had before I got the cancer.” [Female ALL^a^ survivor aged 25 years; 014]	Help survivors to understand their treatment history and details of their survivorship care plan
Relationships with health care providers	Concerned that adult providers will not understand their unique needs. Difficulty moving on from trusted relationships with pediatric providers. Adult health care system is complex and difficult to navigate	“I probably wouldn’t go through the hassle or the time to find a new radiologist in [current location]...the fact that they know me and they know my history does play a big role in it.” [Male Hodgkin lymphoma survivor aged 24 years; 004] “Ideally it would be nice if you could find someone that understands everything you’ve been through. Because I’ve had some effects already happen and they just look at me like oh, that shouldn’t happen to someone your age, but they don’t see what I’ve been through.” [Female ALL survivor aged 21 years; 008] “I don't really know how to describe it but when I see them it’s like aww, it’s like meeting a family member again after a while and so much fun. I can just laugh with them and they become your friends. You know, they...they take care of you and...I’m grateful to them.” [Female ALL survivor aged 23 years; 003]	Strategies for identifying and communicating with new adult health care providers
Relationships with family members involved in health care	Parents provide emotional and logistical support; they want to remain involved because of concerns about their child	“Oh, [my mom’s] totally on top of it. ’Cause when I got sick, I couldn’t handle all the medical stuff. So she did all of it...no matter how much time passes I think that she’s always gonna want to be there. And I don’t mind her being there.” [Female ALL survivor aged 22 years; 016] “I don’t even know what insurance is anymore because my parents just deal with all of that, which is really nice. If I didn’t have my parents dealing with it, I would probably be lost.” [Male ALL survivor aged 21 years; 001]	Communication with parents about ongoing involvement in health care
Emotions about health, follow-up care, and transitions in care	Anxiety during transitions in care; worries about future health problems because of surveillance measures; feeling alone or different from healthy peers	“It was kind of like being thrown out there with nothing to float with. I guess it’s just kind of a shock because I went from almost every day to not seeing a doctor for 3 months. And it was...hard to do that.” [Female non-Hodgkin lymphoma survivor aged 24 years; 011] “I said I don’t want any more chest x-rays. I don’t want them every 4 months at least. Because then I’m just gonna get breast cancer and it’s gonna be a whole nother mess...It’s so awful how...the preventive measures also give you cancer.” [Female Hodgkin lymphoma survivor aged 22 years; 017] “You just feel...kind of alone sometimes. And even afterwards it’s a scary time because, you know, the treatment may have worked or it may not have worked. And kind of in a waiting period it really helps to have some people to talk to that know what you’re going through.” [Female non-Hodgkin lymphoma survivor aged 25 years; 018]	Strategies to cope with emotions about health and follow-up care; peer support
Lifestyle behaviors and life transitions	Focusing on other preventive health behaviors (eg, diet and exercise); prioritizing other important life milestones (eg, pursuing college and career)	“It’s all just prevention at this point, trying to stop complications from happening again or anything like that. So it’s really important to make sure that you’re staying healthy, [to do] extra things that other people your age probably don’t have to do and you do.” [Female AML^b^ survivor aged 21 years; 002] “Sometimes, you know, in the busy lives and as we get older, college and everything, I think we kind of just kind of forget about it, just put it in the back of our minds and then we just kind of ignore it.” [Female ALL survivor aged 20 years; 010]	Encourage healthy lifestyle behaviors and focusing on health in the context of other important life milestones

^a^ALL: acute lymphoblastic leukemia.

^b^AML: acute myeloblastic leukemia.

In the first theme, a lack of knowledge about personal risk for late effects was commonly reported as a barrier to care. Participants who had received a written survivorship care plan found it to be an important tool to educate themselves about their cancer history and risks for late effects that require ongoing monitoring. Participants reported attending follow-up care to monitor for potential late effects.

The second theme centered on how survivors’ relationship with health care providers influenced their beliefs about follow-up care and the likelihood of attending it. Survivors who continued to see their original treatment team for long-term follow-up care cited their personal relationship with these medical providers as a major factor in their obtaining care. Participants commented that they had developed a strong personal bond because of the time spent with the health care providers during treatment and the seriousness of cancer treatment. They trusted their providers and found familiar providers supportive. The survivors also indicated that feeling *cared for* as a person (not just a disease) was important to them. Similarly, the survivors reported that they would be unlikely to continue care if they had to switch providers because of (1) the close relationship developed with the primary oncologist; (2) distrust of a new provider unfamiliar with their treatment history; (3) inconvenience of, and difficulty with, finding a new adult provider skilled in survivorship care; and (4) having to relay complex medical history to a new provider.

The third theme centered on the role of family members, particularly parents, in their medical care. Although emerging adulthood is typically characterized by greater independence from family, participants indicated that their parents were still involved in their follow-up care. Parents had been responsible for their cancer treatment and wanted to remain involved in follow-up care for their own emotional relief, hoping that their child’s cancer would remain in remission. Parents could help by asking questions, remembering what doctors tell them, and providing emotional support during the visit with the survivors. By contrast, attending clinic on their own was a sign of independence and responsibility.

The fourth theme focused on emotions about health, follow-up care, and transitions in care. Attending long-term follow-up care provided reassurance for survivors and their family that their cancer had not recurred. This reassurance could be sought when they experienced symptoms that reminded them of the symptoms leading to their diagnosis, or it could be seen as part of routine follow-up care. Survivors could experience anxiety preceding or during their visits because of triggering of unpleasant treatment memories, but the relief about their health status outweighed their anxiety. Concerns about potential negative effects of screening procedures, including increased risk of developing a second malignancy, were barriers to care. There were also reports of feeling *alone* and *different* from healthy peers. Peer support from others *who know* what they were going through was seen as useful.

The fifth and final theme that emerged related to general preventive health behaviors and a focus on other developmentally appropriate life milestones such as going to college and starting a career. Lack of symptoms or late effects and the belief that one was *in the clear* after a certain amount of time after treatment could reduce motivation to seek care in the context of other life priorities.

### Stage 2: Content and Program Development

In this stage, qualitative interviews with 6 key stakeholders confirmed that the modifiable variables from the SMART model were relevant and important targets for intervention. Specifically, the SMART constructs included knowledge, self-management skills, self-efficacy, relationships and communication, and goals and motivation.

Knowledge, specifically about treatment and potential late effects, was identified as essential for successful self-management by all (6/6, 100%) stakeholders. The AYA survivors noted that their parents were the ones who initially may have received the information about their follow-up plan and potential late effects and that this information needed to be repeated and provided again as they continue into young adulthood. The AYA participants called for a written guideline or map of what they needed to do and when. Similarly, the parent participant echoed this call for more guidance about what care was necessary moving forward. All (3/3, 100%) of the providers mentioned that their respective institutions provided a written survivorship care plan for guideline-concordant follow-up care. They also confirmed how important it was for patients to obtain a copy of this plan and to know where it is and how to access it as well as to bring it with them to future appointments with other providers.

All (6/6, 100%) participants acknowledged that a lack of skills in navigating the logistics of the health care system was a significant barrier to successful self-management. In particular, both the patient participant and 67% (2/3) of the providers specifically mentioned challenges in dealing with insurance. The providers noted the lack of support (including social work, school, and vocation support) for AYA survivors once they leave their pediatric provider. Both the providers and the parent participant acknowledged that AYA survivors may rely on parents for basic logistics of scheduling and keeping appointments, suggesting that they may lack confidence in handling these tasks themselves. When asked what topics would be a priority for a self-management program, the logistics of navigating the health care system was listed by 83% (5/6) of the participants.

Communication, both with providers and with parents and family members, was another barrier to successful self-management and follow-up care confirmed by participant responses. The AYA survivors described feeling fearful of meeting with new providers because they worried that not all providers would *understand them* and are *not insightful of specific symptoms or feelings* that may be unique to survivors. Of the 3 providers, 1 (33%) echoed sentiments about lack of effective communication and stated as follows:

I don’t think survivors are always equipped especially at that age to advocate for themselves...that’s maybe a skill that can be developed—how to better advocate for themselves in the health care system, particularly when you’re talking with your doctor.

The AYA survivors also mentioned difficulty with talking with their parents about their disease or about taking over some of the tasks related to follow-up care.

Finally, all (6/6, 100%) stakeholders mentioned the importance of learning about other healthy behaviors (both for disease prevention and health maintenance). The AYA survivors described the difficulty of coping with other life events such as school or finding a job in conjunction with maintaining healthy behaviors. The providers echoed this sentiment, reporting that many times survivors will focus on cancer follow-up while excluding other important healthy behaviors, including tobacco use, safe alcohol use, and sun protection behaviors. All (6/6, 100%) felt that it would be appropriate to provide education about maintaining *general health* within the context of a self-management intervention. When presented with the idea of providing educational content through technology along with a peer mentor, the stakeholders were receptive to the approach. All (6/6, 100%) stakeholders reported that the use of technology such as video calling and SMS text messaging would appeal to survivors. Several (4/6, 67%) stakeholders suggested that mentors should be outgoing advocates; they also suggested matching by diagnosis, if possible, but recognized that finding other things in common would be important for building a relationship. The stakeholders did not identify any new topics outside of the model and themes from stage 1; therefore, we determined that there was thematic saturation after these 6 key stakeholder interviews and moved to creating prototype content for usability testing.

Guided by these results and the SMART framework, we developed a prototype of the web-based self-management skills and peer-mentor intervention. We proposed that the intervention would consist of two components: (1) web-based educational modules to improve self-management skills (using Rutgers Canvas) and (2) a peer mentor to provide support and facilitate engagement with the modules. The web-based self-management modules encompass five key areas: (1) understanding treatment history and the survivorship care plan, (2) managing the logistics of health care, (3) negotiating family involvement in survivorship care, (4) dealing with emotions about survivorship health and follow-up care, and (5) staying healthy in the context of life transitions. Given the amount of self-management education, we decided that a website would be optimal for housing the information and delivering it consistently to all participants. Table 3 presents the proposed content, the SMART constructs targeted by each module, and an illustrative quote from a key stakeholder affirming the relevance of the content. We proposed that the peer mentor would meet with the participant to review the content of the module, discuss how the participant could apply the content, and offer support. Mentors would also remind participants to complete the modules before their meeting.

**Table 3:** Proposed modules based on formative research and content development interviews.

Proposed module	Module content	SMART^a^ constructs targeted	Supportive quote
1. Understanding treatment history and survivorship care plan	Name diagnosis, treatments received, and risks for late health effects Understand your treatment history Risk of late effects Obtain (if needed) and store survivorship care plan	Knowledge Goals and motivation	“I think just like knowing about what I could see in the future, what’s common, what’s not. What’s common with like the treatment I received? And things like that, I think that like mostly what I’m concerned about.” [AYA^b^-1]
2. Managing your health care	Review self-management tasks (eg, make appointments and obtain screenings) Establish and maintain relationship with primary care physician Logistics of insurance and health care tasks Identify barriers to obtaining care and problem solve Review motivation and confidence to assume responsibility for care	Self-management skills Self-efficacy Relationships and communication Goals and motivation	“I think most of the AYAs have a sense of how the health care system runs but I think even just the basic logistics of who do you call to schedule an appointment. You know who do you ask for, for what resource? Where do you get your medications? Things that their parents take care of at a very detailed level, but they sort of understand what the process is because their parents have been doing it for them all along.” [HP^c^-1]
3. Negotiating family involvement in your care	Discuss challenges of parents who do not relinquish control and issues related to communication skills Discuss supportive ways to include family	Relationships and communication	“It more or less started when I went to the doctors and I was talking to them, it was before I went to college. And I guess after that day my mom kind of realized you know she’s older. I’m able to sign my own forms. I was able to be at the office by myself. She didn’t necessarily have to go to the office so when the doctor started directing the questions and the suggestions to only me and not my mom as well it kind of clicked that hey you know I’m going to have to be an adult. I’m going to have to start taking charge of my own health.” [AYA-2]
4. Dealing with emotions about your health and follow-up care	Coping with uncertainty of future health Communicating with providers and families about adult-oriented health care	Self-management skills Relationships and communication	“Well, I think that one of the major things is just worrying about her health. You know and hoping that everything still goes forward in the right direction.” [P^d^-1]
5. Staying healthy in the context of life transitions	Recognize that health must be maintained in the context of other important life transitions (eg, education, career, and relationships) Skills and resources for healthy diet, exercise, sexual health, fertility, education, and career Identify value in prioritizing health	Goals and motivation Self-management skills Self-efficacy	“My goal is that if you take good care of your body and make wise choices your survivorship will be no different than your peers. By thinking about that you know smoking you know or doing drugs or not wearing your seat belt when you’re in the car, all of those things are potential factors that could end your life sooner than, per se, your peers so based on all of the therapy that you received you’re fortunate that you’re here today and what can we do to make sure that your lifelong health is protected?” [HP-2]

^a^SMART: social-ecological model of adolescent and young adult readiness for transition.

^b^AYA: adolescent and young adult (survivor).

^c^HP: health care provider.

^d^P: parent.

### Stage 3: Content Refinement

All (4/4, 100%) participants found the content in the modules to be relevant and important for managing their own health. The AYA survivors found the website easy to use and reported that the instructions for progressing through the program were clear. In general, there was a desire for more participative content, including animated videos and tailored quizzes. The survivors indicated a preference for information distribution through infographics and illustrations versus a written *e-book–*type format. Participants made specific recommendations for word changes to improve readability. There were requests for links to additional resources, including support groups, scholarships, and career resources. Of the 4 survivors, 1 (25%) suggested adding a group discussion forum for all participants. Participants also indicated that they would be likely to access the website on both a computer as well as their mobile phones, and as such, the website should be tailored for mobile use. In response to these suggestions, we added an animated *how-to* video, quizzes for each module, and changed some text to improve readability. We also added a specific *resources* section covering the array of topics suggested. We decided not to add a group discussion forum at this time, given that it would require resources to moderate and add complexity to the intervention. However, we decided we would add a section where mentors could upload a picture and brief biography to share their stories. Our goal was to incorporate as many suggestions as possible with our limited resources so that we could move quickly to a feasibility trial. Sample screenshots of the revised prototype are presented in Multimedia Appendix 1.

When asked specifically about their preference for mode of contact with the peer mentor (ie, videoconference, telephone, SMS text message, and social media), all (4/4, 100%) survivors expressed a preference for communicating with a peer mentor through videoconference or telephone and SMS text message rather than social media. They were receptive to the idea of working with a mentor. Of the 4 participants, 1 (25%) suggested that it would be helpful for the mentor to have received the same diagnosis as they, but another (1/4, 25%) suggested that shared interests or mentor training would be important to successfully initiate a relationship. Although SMS text messaging offers convenience, we felt that video calling would be optimal to build a relationship. With our participants’ preferences in mind, we decided that we would introduce the peer mentor–participant pairs through secure SMS text message and then the peer mentor would be responsible for initiating 6 videocalls with their mentee. The first call would focus on rapport building, whereas the remaining calls (5/6, 83%) would focus on discussing and applying the content of the 5 web-based modules (ie, 1 module per call). We also plan to train and supervise mentors closely to facilitate positive relationships.

## Discussion

### Principal Findings

Childhood cancer survivors are a growing population with a demonstrated need for developmentally appropriate and evidence-based interventions to improve their transition readiness and self-management skills. The goal of this study was to develop a theory-based intervention to improve transition readiness of AYA survivors.

In the first stage of the study, the formative stage, we identified barriers and facilitators to AYA survivors receiving risk-based care, including lack of knowledge about personal risk for late effects, relationships with their medical care team and parents, emotional reassurance about health and remission status, and motivation to remain healthy and pursue normative developmental milestones. Participants reported that their parents remained involved in their follow-up care. The survivors discussed the emotional challenge of being *different* from healthy peers, with the implication that getting support from peers who had successfully navigated the transition and understood what it is like to be a survivor could be helpful. These results align well with the broader literature on barriers to recommended survivorship care, particularly during and after the transition to young adulthood [29,30]. Combined with the existing literature, our results suggested that a skills-based self-management intervention that also incorporates social support from a peer could address the needs of this population.

In the second stage of the study, key stakeholders (ie, AYA survivors, parent, and providers) confirmed that our identified self-management topics (from stage 1 formative research and the SMART framework) were relevant and important components for an intervention. The stakeholders highlighted the need for increased knowledge about the risk of late effects, enhanced skills for self-management and health care system navigation, strategies for communicating with health care providers and family members involved in care, coping with the emotions of long-term survivorship care, and incorporating healthy behaviors into their everyday lives. These topics were incorporated into a series of web-based modules created to help improve self-management skills. The use of peer mentoring alongside the self-management skills modules is intended to provide support and advice regarding emotional and practical barriers to transition from someone with similar experience. In addition, a peer mentor may serve as a supportive accountability agent, facilitating participants’ engagement with the web-based modules. The program was designed such that the mentee participant would complete 1 module per week and review it with their peer mentor.

The web-based modules were well received by the AYA survivors in stage 3 usability testing and were seen as helpful and informative for managing their care. Suggestions to improve the modules were primarily to make them more participative to increase usability and engagement. The AYA survivors were receptive to having a peer mentor. Mentoring has been found to be an acceptable method of intervention among other AYAs with chronic illnesses such as irritable bowel disease, juvenile arthritis, chronic pain, and juvenile diabetes [31-34]. The AYA survivors expressed a preference for using SMS text messaging, telephone calls, or videoconference to communicate with their mentor. Of the 4 AYA survivors, 1 (25%) expressed a preference for being *matched* with their mentor based on cancer type. This preference aligns with the theory of social networks, which proposes that social network ties with those who have direct personal experience with a life event or *experiential similarity* are more likely to offer specialized health-related informational support. Peer survivors, particularly those who have had similar diagnoses and treatments, can offer informational and emotional support through empathic understanding of the concerns of AYA survivors regarding health care self-management, serve as role models, and provide advice and encouragement as AYA survivors take greater responsibility for managing their health care [35,36]. Using a peer survivor mentor is novel compared with existing interventions [17]. However, there may be challenges in recruiting mentors with the same diagnosis, given the relative rarity of some diagnoses.

### Limitations

The limitations of this study include reliance on a small sample to move quickly through the stages of development. Although we believed that it was important to recruit beyond our own local clinic catchment area, advertisements through social media and web-based platforms yielded a sample that was primarily female and lacked racial and ethnic diversity, which could limit whether our proposed intervention meets the needs of unrepresented groups. The use of telephone interviews allowed recruitment of individuals from different geographic locations where survivorship care may be different. Telephone interviews (vs in-person interviews) have advantages and disadvantages; telephone interviews may increase disclosure of information by respondents but prohibit observation of nonverbal cues [37]. The SMART model and existing literature were used to form the interview guides and develop the intervention. This gives the content strong theoretical underpinnings but may have limited creativity because our questions were aligned with the framework rather than more open-ended creative design exercises. We also limited our inclusion criteria to AYA survivors currently aged 18 to 25 years because they are legally responsible for their health care and this period is a common time of transfer from pediatric-centered to adult-centered health care. However, transition is a process that should start earlier in adolescence, and our design process did not capture the input of those who were in the earlier stages of the process.

### Conclusions

In conclusion, AYA survivors of childhood cancer are interested in a self-management and peer-mentoring intervention that may improve their confidence and skills to manage their own care. Incorporating AYA survivors into this formative work helped us to confirm theoretically important targets for the intervention and make design decisions in line with our target users’ preferences. Although our focus was on AYA survivors of childhood cancer, the barriers and essential intervention topics we identified may be relevant for AYAs with other chronic health conditions because a recent systematic review found that relationship, access to care, insurance, knowledge, and self-management skill barriers transcend illness conditions [38]. Future research is needed to understand the feasibility and acceptability of the proposed intervention. The next step is to conduct a single-arm trial to determine the feasibility and acceptability of the proposed intervention among AYA survivors of childhood cancer.

## Appendix

Multimedia Appendix 1 Sample screenshots of the prototype educational modules.

## Abbreviations

AYA: adolescent and young adult
SMART: social-ecological model of adolescent and young adult readiness for transition
