# Supplementary material for: Development of a Self-management and Peer-Mentoring Intervention to Improve Transition Readiness Among Young Adult Survivors of Pediatric Cancer: Formative Qualitative Research Study
Source: JMIR Form Res. 2022 Aug 3;6(8):e36323. doi: 10.2196/36323 (PMC9386586; doi:10.2196/36323)

Multimedia Appendix. Sample screenshots of the prototype educational modules.

This is a Multimedia Appendix to a full manuscript published in the JMIR Form Res. For full copyright and citation information see http://dx.doi.org/10.2196/jmir.36323

Homepage (included Introduction and overview of all modules and resources).

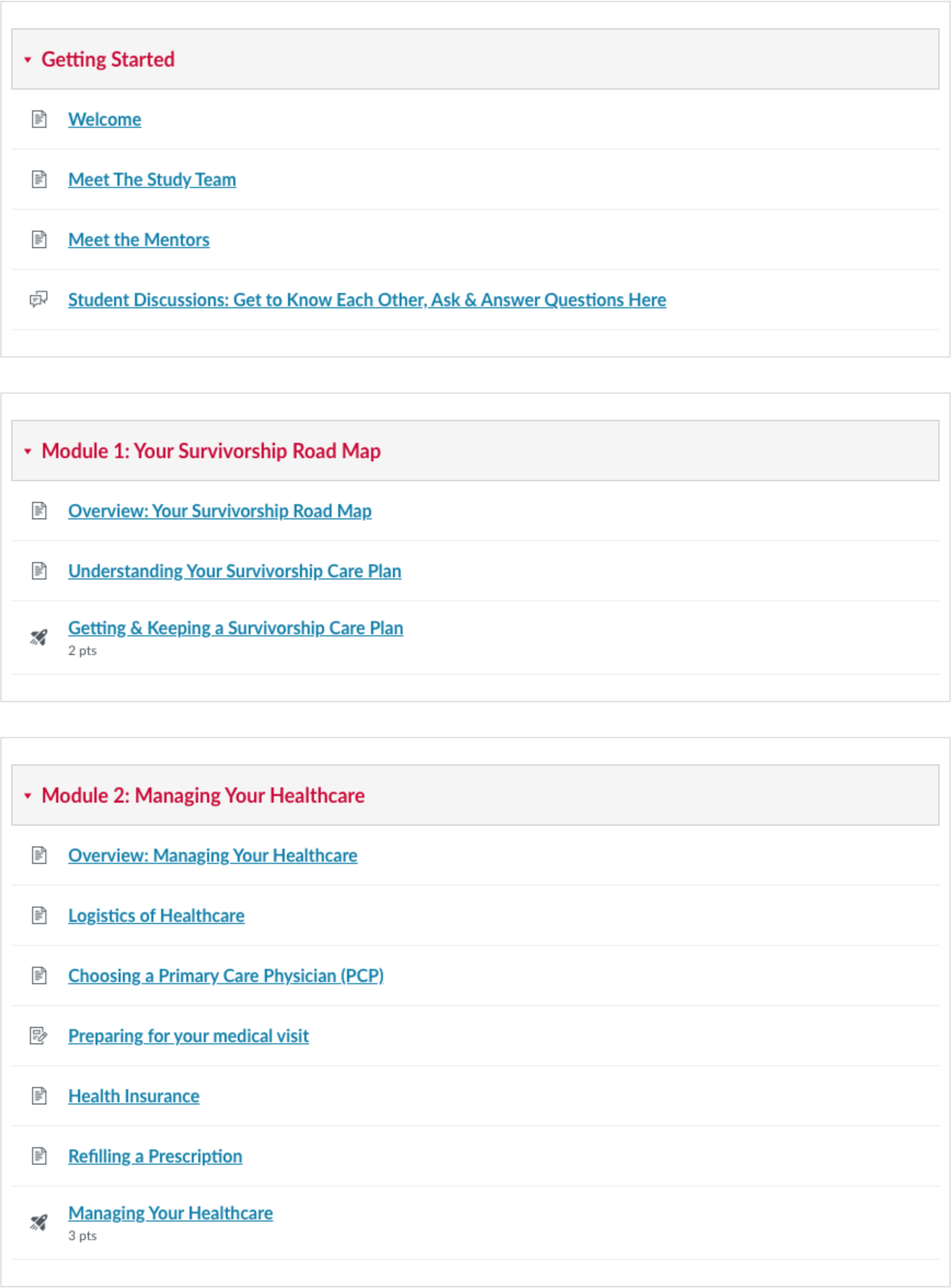

Module 3: Family Involvement

- Overview: Family Involvement
- Negotiating Family Involvement in Your Care
- Family Involvement in Care

Module 4: Dealing with Emotions about Your Health and Follow-Up Care

- Overview: Emotions
- Dealing with Emotions about Health and Follow-Up Care
- Problem-Solving
- Communication Skills

Module 5: Staying Healthy in the Context of Life Transitions

- Overview: Staying Healthy
- Stress Management
- Time Management
- Sleep
- Healthy Eating
- Exercise
- Substance Use
- Sexual Health
- Sun Protection
- Staying Healthy
  2 pts

Resources

- General Resources
- Long-Term Follow-Up Care
- Financial Resources
- School Resources
- Disability
- Female Specific Resources
- Career

Sample Page from Module 1:

Overview: Your Survivorship Road Map

Your Survivorship Road Map

Introduction

This module provides an overview of long-term follow-up care after cancer treatment is over. Understanding your health history is critical for maintaining your health. The goal of this module is to help you figure out how much you understand about your health and get any information you need.

Learning Objectives

Upon completion of this module, you should be able to:

1. Name your diagnosis, treatments received, and risks for late health effects.
2. Obtain (if needed) and store your survivorship care plan.
3. Identify any health screenings you need going forward and how often you need them.

Sample Quiz Assessing Knowledge (from Module 2):

Question 1 — 1 pts

Test your knowledge of health insurance lingo.

Coinsurance	[Choose]
Deductible	[Choose]
Copayment	[Choose]
Premium	[Choose]
Lifetime Limit	[Choose]
Pre-existing Condition	[Choose]
Essential Health Benefits	[Choose]
Preventive Services	[Choose]

Sample Quiz to Encourage New Skill (from Module 4):

Question 5 — 1 pts

What communication skill(s) do you plan to work on?

- [ ] Asking open-ended questions
- [ ] Avoiding polarizing words (never, always)
- [ ] Practicing good non-verbal skills
- [ ] Using "I feel" statements to share how you are feeling with someone else in a non-accusatory way
- [ ] Taking the other person's point of view
- [ ] Listening first
- [ ] Staying calm during a conflict

Sample animated “how-to” video (from Module 2):

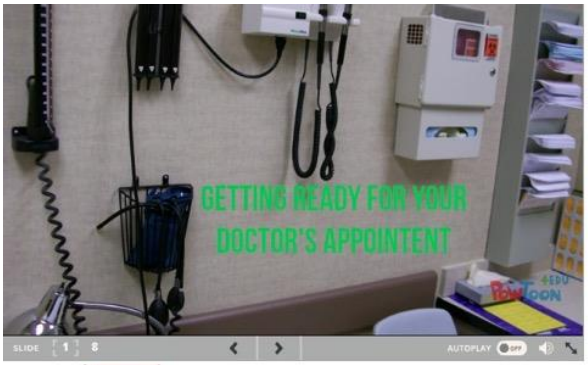

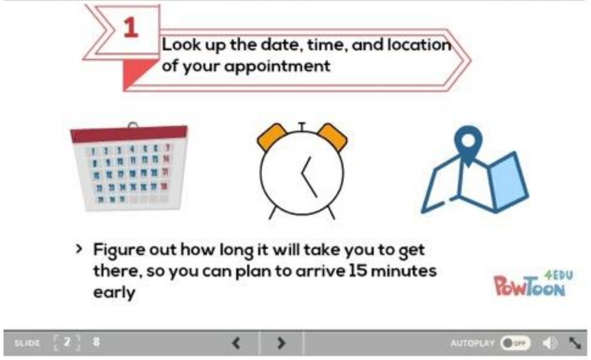

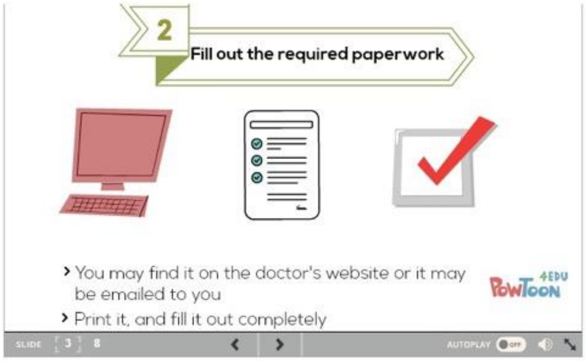

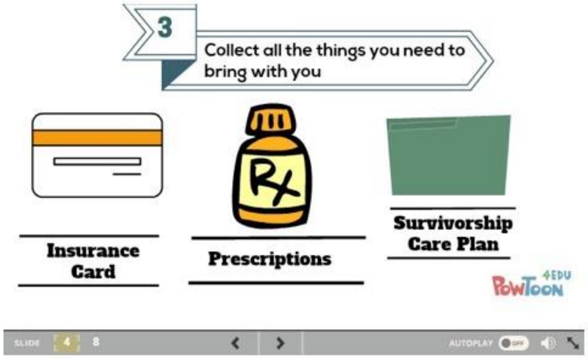

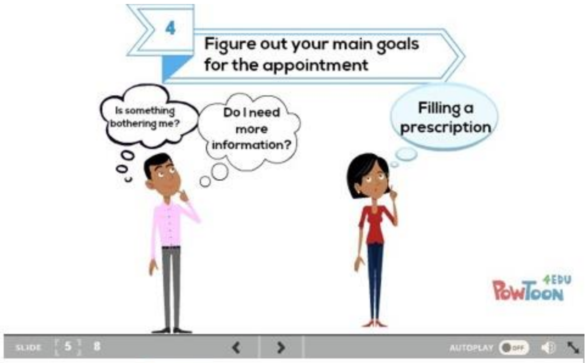

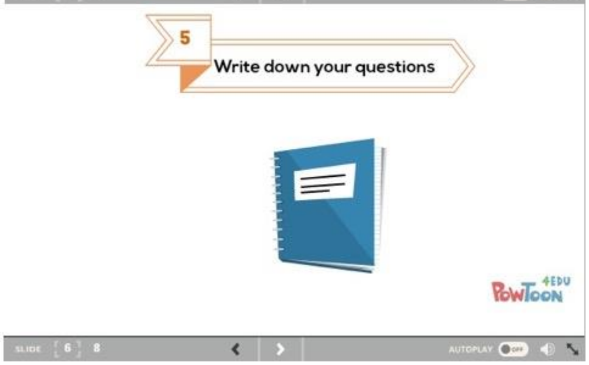

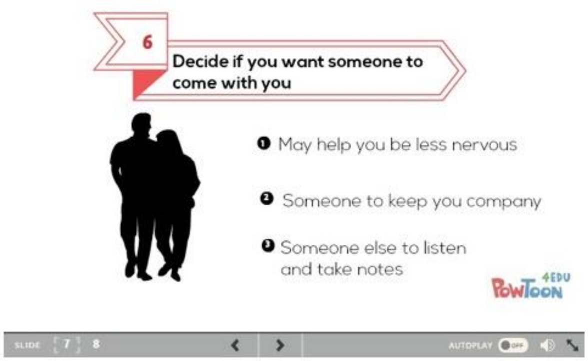

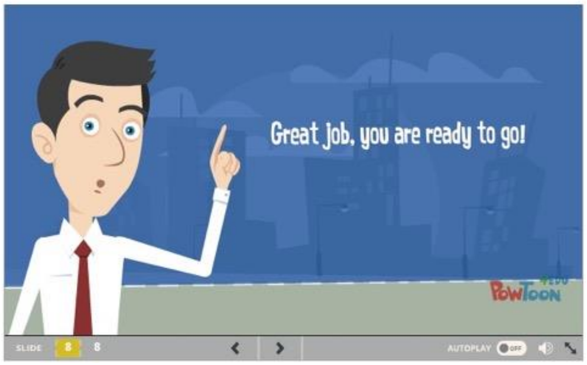

Supplement: Multimedia Appendix 1 [file formative_v6i8e36323_app1.pdf]
